# Therapeutic use of carbohydrate-restricted diets in an autistic child; a case report of clinical and 18FDG PET findings

**DOI:** 10.1007/s11011-018-0219-1

**Published:** 2018-04-11

**Authors:** Iwona Żarnowska, Beata Chrapko, Grażyna Gwizda, Anna Nocuń, Krystyna Mitosek-Szewczyk, Maciej Gasior

**Affiliations:** 1 Department of Pediatric Neurology, Gębali 6, 20-093 Lublin, Poland; 2 Department of Nuclear Medicine, Jaczewskiego 8c, 20-090 Lublin, Poland; 3 Department of Pediatric Otolaryngology, Phoniatry and Audiology, Gębali 6, 20-093 Lublin, Poland; 4 Department of Pharmacology and Physiology, Drexel University College of Medicine, Philadelphia, PA USA

**Keywords:** Ketogenic diet, Autism, 18FDG PET, Glucose metabolism

## Abstract

The ketogenic diet (KD) is a high-fat, adequate-protein, and low-carbohydrate diet that has been used successfully in the treatment of refractory epilepsies for almost 100 years. There has been accumulating evidence to show that the KD may provide a therapeutic benefit in autism spectrum disorders, albeit by a yet-unknown mechanism. We report a case of a 6-year-old patient with high-functioning autism and subclinical epileptic discharges who responded poorly to several behavioural and psychopharmacological treatments. The patient was subsequently placed on the KD due to significant glucose hypometabolism in the brain as revealed by an 18FDG PET. As soon as one month after starting the KD, the patient’s behavior and intellect improved (in regard to hyperactivity, attention span, abnormal reactions to visual and auditory stimuli, usage of objects, adaptability to changes, communication skills, fear, anxiety, and emotional reactions); these improvements continued until the end of the observation period at 16 months on the KD. The 18FDG PET, measured at 12 months on the KD, revealed that 18F-FDG uptake decreased markedly and diffusely in the whole cerebral cortex with a relatively low reduction in basal ganglia in comparison to the pre-KD assessment. It warrants further investigation if the 18FDG PET imaging could serve as a biomarker in identifying individuals with autism who might benefit from the KD due to underlying abnormalities related to glucose hypometabolism.

## Introduction

The ketogenic diet (KD) has been utilized for nearly 100 years to effectively manage refractory epilepsy, especially in children and adolescents (Kossoff et al. 2016; Wheless 2008). There are several variations of the KD. Three were applied in the present study: the classic KD, modified Atkins diet (MAD), and low glycaemic index treatments (LGIT). The classic KD is a very strict, high-fat, low-carbohydrate, adequate-protein and vitamin-supplemented diet with meals distributed evenly throughout the day. Calories are calculated individually for each patient with a defined ketogenic ratio (grams of fats: grams of proteins+carbohydrates) from 4:1 to 2:1 in every meal. More recently, less restrictive versions of this classic KD have been introduced to increase the diet’s palatability and adherence (e.g., MAD and LGIT). Both the MAD and LGIT are similar to the classic KD in that they both are high fat/low carbohydrate diets and require thorough medical supervision and vitamin/mineral supplementation. The main differences between the classic KD and the modified diets are the relative recommended proportions of specific macronutrients and the strictness of adhering to the feeding regimen. The alternative diets allow more carbohydrates and proteins, and calories are less strictly monitored; they are also less restrictive in terms of meal distribution throughout the day and carbohydrate variability. Evidence from clinical studies suggests that these alternative diets may provide therapeutic benefits similar to the classic KD (Kossoff et al. 2016).

The KD reduces seizure burden and improves development and behaviour, specifically attention and social functioning (Pulsifer et al. 2001). The KD has also proven effective for individuals with glucose transporter type 1 (GLUT-1), pyruvate dehydrogenase deficiencies, and respiratory chain defects by providing an alternative source of acetyl-CoA. The diet is now being considered for diverse neurological indications beyond epilepsy (Gasior et al. 2006; Stafstrom and Rho 2012).

Autism spectrum disorders (ASD) are heterogenic neurodevelopmental disorders characterized by social communication impairments, the presence of restricted interests and repetitive behaviours, and/or sensory over-responsivity (American Psychiatric Association 2000). ASDs have heterogeneous etiology, clinical presentations, and associated symptoms. To date, an ASD diagnosis is based solely on clinical criteria; there are no biomarkers that can be used to diagnose the condition. Symptoms of ASD are frequently co-morbid with epilepsy or subclinical epileptic discharges (Hara 2007), and these cases are often associated with more severe ASD symptoms and higher degrees of intellectual disability (El Achkar and Spence 2015; Tuchman and Rapin 2002). Although the molecular mechanisms underlying the disorder are not fully understood and effective treatments are still lacking, there is accumulating evidence from pre-clinical studies that supports the efficacy of the KD in treating epilepsy and core symptoms of ASD (Ahn et al. 2014; Ruskin et al. 2013, 2017; Verpeut et al. 2016). The KD has been shown to enhance mitochondrial function through multiple mechanisms and affect additional molecular targets that may address symptoms and comorbidities of ASD (Cheng et al. 2017). Additionally, one clinical pilot study and several case reports indicated that children with ASD treated with the KD showed decreased seizure frequencies and improved behaviour, learning abilities, cognitive functioning, and social skills (Evangeliou et al. 2003; Herbert and Buckley 2013; Kinsman et al. 1992).

Neuroimaging studies have demonstrated structural, functional, and neurochemical differences in the areas of the so-called “social brain” (i.e., the amygdala, superior temporal sulcus, orbitofrontal cortex, medial prefrontal cortex, and insula), in individuals with ASD when compared to controls (Ventola et al. 2013; Zurcher et al. 2015). However, neuroimaging studies using MRI have provided mixed and at times contradictory results and often failed to provide a direct link to the clinical symptomatology of the disorder (Hernandez et al. 2015; Li et al. 2017).

A non-invasive positron emission tomography (PET) with 18 fluoro-deoxyglucose (18FDG PET) of the resting brain is an imaging technique that measures brain glucose metabolic rate, summed over a 35-min FDG radiotracer uptake period. In ASD, fMRI, PET, and post mortem brain analyses have shown that the amygdala, superior temporal sulcus, orbitofrontal cortex, fusiform gyrus, insula, anterior cingulated gyrus, and basal ganglia could be important sites of functional alteration (Hernandez et al. 2015; Ventola et al. 2013; Zurcher et al. 2015). Therefore, we hypothesize that these brain structures would show glucose metabolic rate changes in patients with ASD, which may be influenced by the KD.

This case report reviews the history of a patient with high functioning autism and subclinical epileptic discharges who had poor response to behavioural and psychopharmacological treatments. The patient was subsequently placed on the KD due to significant glucose hypometabolism in the brain as revealed by 18FDG PET.

## Case report

The patient is a six-year-old Caucasian boy, the younger in a family of two children (his sister was born with sensorineural hearing loss due to a common mutation of GJB2:35delG and received cochlear implants; her speech and development were normal after long-lasting successful rehabilitation). The patient was born after a normal, full-term pregnancy, delivered via normal, vaginal delivery at 40 weeks, with APGAR scores of 10/10. Early development was affected by recurrent upper respiratory tract infections frequently treated with antibiotics. At the age of 2, he was developmentally on target in motor and cognitive skills, used language for communication, and displayed normal interests, social activities, and behaviours that were appropriate for his age. At the age of 3, his parents noticed speech regression and behavioural deteriorations with unexplained irritability, tantrums, aggression, impaired reciprocal social interactions with limited and stereotyped interests and activities, and unusual responses to visual and auditory stimuli (i.e., moderate impairment to sort out multiple objects in visual scene, an inability to discern the orientation between objects in space resulting in fine motor dysfunction, and anxious reactions to normal hearing stimuli). Consequently, the patient was diagnosed with early childhood autism, mental retardation, and attention-deficit hyperactivity disorder (ADHD) after psychological and psychiatric evaluations, using the Diagnostic and Statistical Manual of Mental Disorders, 4th Edition, Text Revision (DSM-IV-TR) criteria (American Psychiatric Association 2000). The diagnoses allowed him to access a specialized programme for children with autism in a government-funded early intervention programme. He has since participated in behavioural treatments organized in a kindergarten for autistic children. At the age of 4, the patient underwent a detailed paediatric and neurologic evaluation which did not reveal any clinically meaningful abnormalities. The 1,5 T MRI brain scans were normal. The sleep-phase electroencephalogram (EEG) revealed bilateral, synchronous and asynchronous centro-temporal spikes and spike-wave complexes; however, epileptic-like events had never been observed in the patient’s behaviour. Due to increased behavioural problems and ongoing EEG abnormalities, a pharmacologic treatment with valproic acid was introduced and maintained for 12 months; but it had no effect on behaviour or the EEG. Concomitantly, at the age of 5, methylphenidate, an ADHD medication, was introduced and maintained for 6 months; however, it did not affect attention span while significantly worsening core symptoms of autism. The patient was re-evaluated at the age of 6 by a child neurologist with no new findings.

Genetic testing for Fragile X syndrome was negative. A genome-wide search for imbalances as causes of ASD was also negative. However, a PET examination was performed that revealed a marked decrease in 18FDG glucose metabolism in temporal areas of the brain and within basal ganglia and cerebellum. These findings prompted the introduction of KD therapy as recommended by the International Ketogenic Diet Study Group (Kossoff et al. 2009).

At the age of 6, the patient scored 43 points on the adapted Childhood Autism Rating Scale (CARS). According to this scale, scores of 34 and over at the age of 6 indicate severe cases of ASD (Schopler et al. 1980). The patient had difficulty in controlling and maintaining his emotions, and was over-active. He also suffered from many auditory phobias and object obsessions, had difficulty with social interactions, and obsessively asked questions; however, nonverbal communication was normal. Of note, children with these types of symptoms are often diagnosed with high-functioning autism. The intellectual development of the patient, as measured by the Wechsler Intelligence Scale for Children- revised (WISC-R) at the age of 6 years and 1 month, revealed a Full Scale IQ of 82 - a border-line score for being considered intellectually disabled (Wechsler 1974); the Performance Scale IQ of 62 was lower than average, but the Verbal Scale IQ of 102 was average.

The KD was introduced at the age of 6 years and 1 month, soon after the neuropsychological evaluation. Eligibility for the KD treatment was in concordance with the generally accepted guidelines (Kossoff et al. 2009). The recommended diet was a classic KD with a 2:1 ratio of fats to proteins + carbohydrates that was slowly introduced at home over a 4-week period. The patient achieved optimal ketosis, where his β-hydroxybutyrate level in serum was over 4.0 mmol/l and his stable blood glucose ranged from 65 to 78 mg/dL. After 1 month on this classic KD, the diet was switched per the parents’ request to a MAD to allow for 15–18 g of carbohydrates daily and more proteins; the MAD was maintained for the next five months. While on the MAD, urine ketones were moderate (over 40 mg/dL) consistently and β-hydroxybutyrate level in serum was over 2.0 mmol/l; the diet was well-tolerated. After five months on the MAD, the patient refused the required fat intake and, in accordance with dietary recommendations, he was placed on the LGIT. The LGIT limited carbohydrate intake to 40–60 g per day (approx. 10% of daily calories) and restricted carbohydrates from foods with known high glycemic index scores. After the introduction of the LGIT, the patient’s ketones were still detectable in urine at low to moderate levels (5–15 mg/dL). The patient also received vitamin and mineral supplements according to the recommended daily allowance for his age (Kossoff et al. 2009). Metabolic outcome was monitored at 1, 3, 6, and 12 months after diet initiation of the classic KD. Throughout the observation period, no clinically meaningful changes in blood laboratory parameters (including those relevant for liver and kidney functions, electrolytes, cholesterol, and lipid profiles) were observed. The levels of vitamin D as well as free and total carnitine were normal. No clinically meaningful abnormalities were also evident in urinalysis.

Of note, improvements in clinical outcomes were observed as early as 1 month after the classic KD initiation. They first included behaviour normalization with less hyperactivity and aggressive behaviours. The patient adhered to the diet according to his dietician’s recommendation and no side effects of the KD were observed, as mentioned earlier. Throughout the whole dietary treatment time, no psychopharmacological treatment was needed.

Another psychological evaluation was performed when the patient was on the LGIT at the age of 7 years and 5 months. This occurred 16 months after the initial classic KD implementation. During this second evaluation, the patient scored 27 points on the CARS scale - an improvement of 16 points relative to his first evaluation. On this scale, scores of 28–36 indicate mild to moderate cases of ASD, while lower (< 28) scores indicate minimal-to-no symptoms cases. Some of the improvements on the scale included: reduced hyperactivity, fear, anxiety, emotional reactions, and abnormal visual/auditory reactions, increased attention, improved use of objects, adaptability to changes, and communication abilities. The patient’s behavior had notably changed, especially in regard to his emotions. His communication abilities also improved. The intellectual development of the patient, as measured by the WISC-R at the age of 7 years and 5 months, also improved his Full Scale IQ increased from 82 to 99 (now considered average), Verbal Scale IQ increased from 102 to 113 (now considered average), and Performance Scale IQ increased from 62 to 83 (now considered slightly lower than average).

## 18FDG PET findings

PET of the brain was performed before treatment (baseline study) and 11 months later on the KD (follow-up study), using a dedicated system Biograph mCT S (64)-4R (Siemens, Germany), one hour after injection of 83 MBq of 18F-FDG. The patient fasted on the day of examination. PET acquisition time was 10 min. The reconstruction method as follows: TrueX+TOF (ultraHD-PET) 4 iterations, 21 subsets, filter Gaussian FWHM 2.0 mm, image size 400 × 400 (matrix), zoom 2,0. Images were evaluated on a dedicated workstation equipped with Siemens Scenium software allowing for a quantitative stereotactic 3-dimensional analysis. Using a template of normal subjects, images were normalized to the whole brain and standard deviations (SD) from the norm were calculated. For the semi-quantitative analysis of 18F-FDG uptake in the brain regions, the mean standardized uptake values (SUVs) were calculated by the software, using the following formula: radiotracer concentration in MBq/g divided by the activity injected per gram of the body weight (MBq/g).

**Fig. 1:**
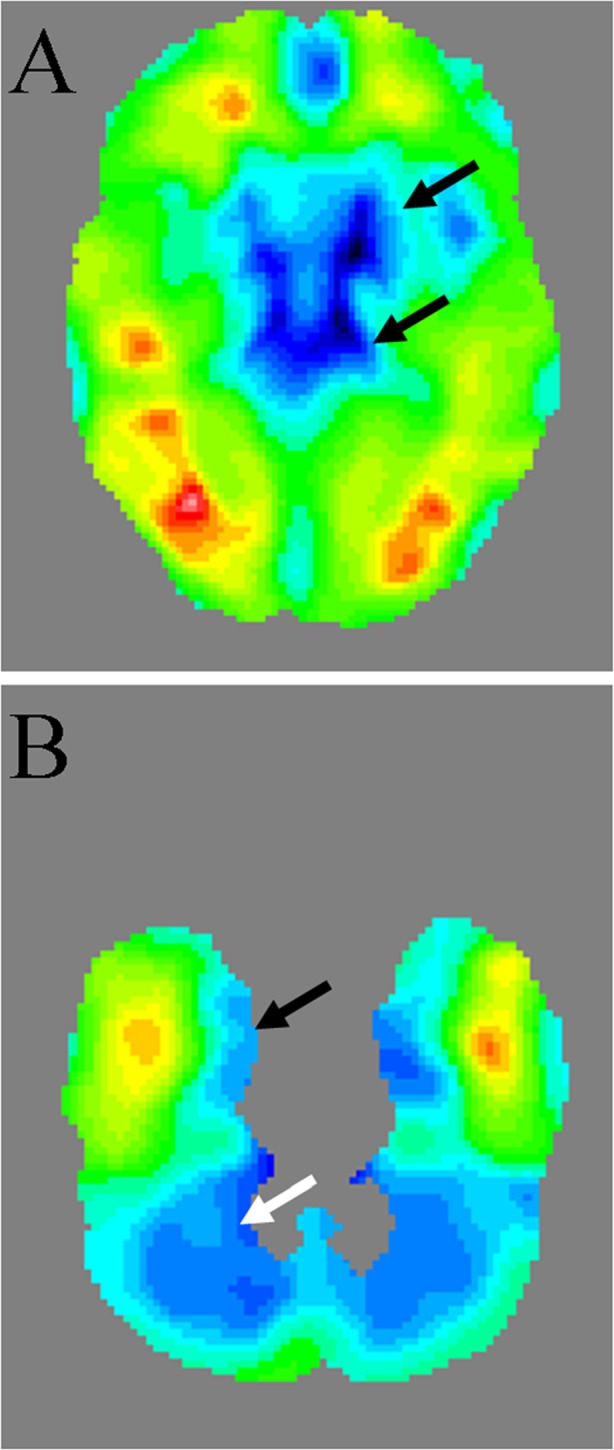
Baseline (before starting KD) 18FDG PET, distribution of standard deviations from the template. **A** decreased glucose uptake in the basal ganglia– arrows (SD– in the lenticular nucleus– right 3.1, left 3.0, in the thalamus– right 5.3, left 6.5). **B** decreased glucose uptake in the mesial temporal lobes– black arrow and cerebellum– white arrow (SD- in the mesial temporal lobe 18FDG PET– right 3.5, left 5.0, in the cerebellum– right 4.7, left 4.9). SD– standard deviation

**Fig. 2:**
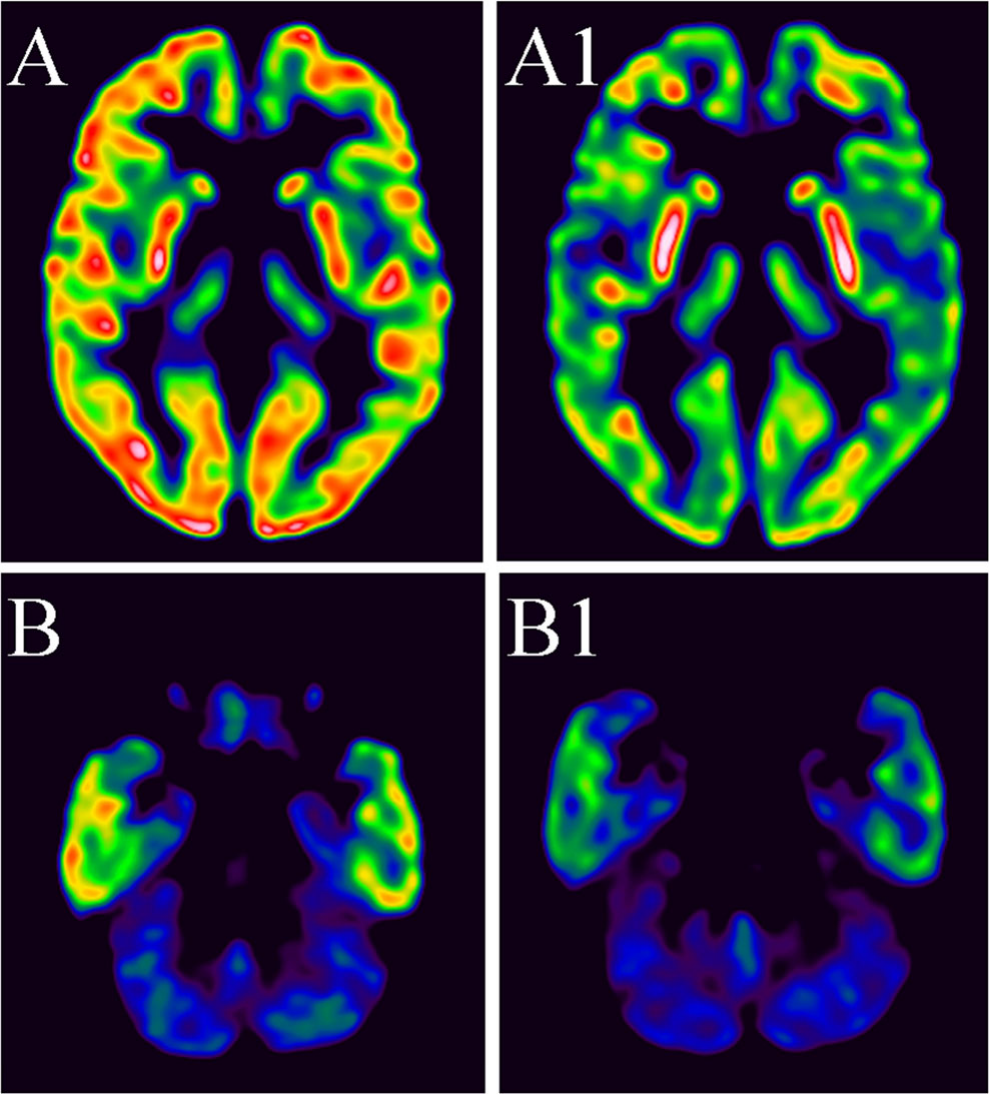
18FDG PET transaxial slices: Baseline images (ie before starting KD) at the level of basal ganglia (**A**) and at the level of the cerebellum (**B**). Decreased activity in the brain in control images at the level of basal ganglia (**A1**) and on the level of the cerebellum (**B1**)

**Fig. 3:**
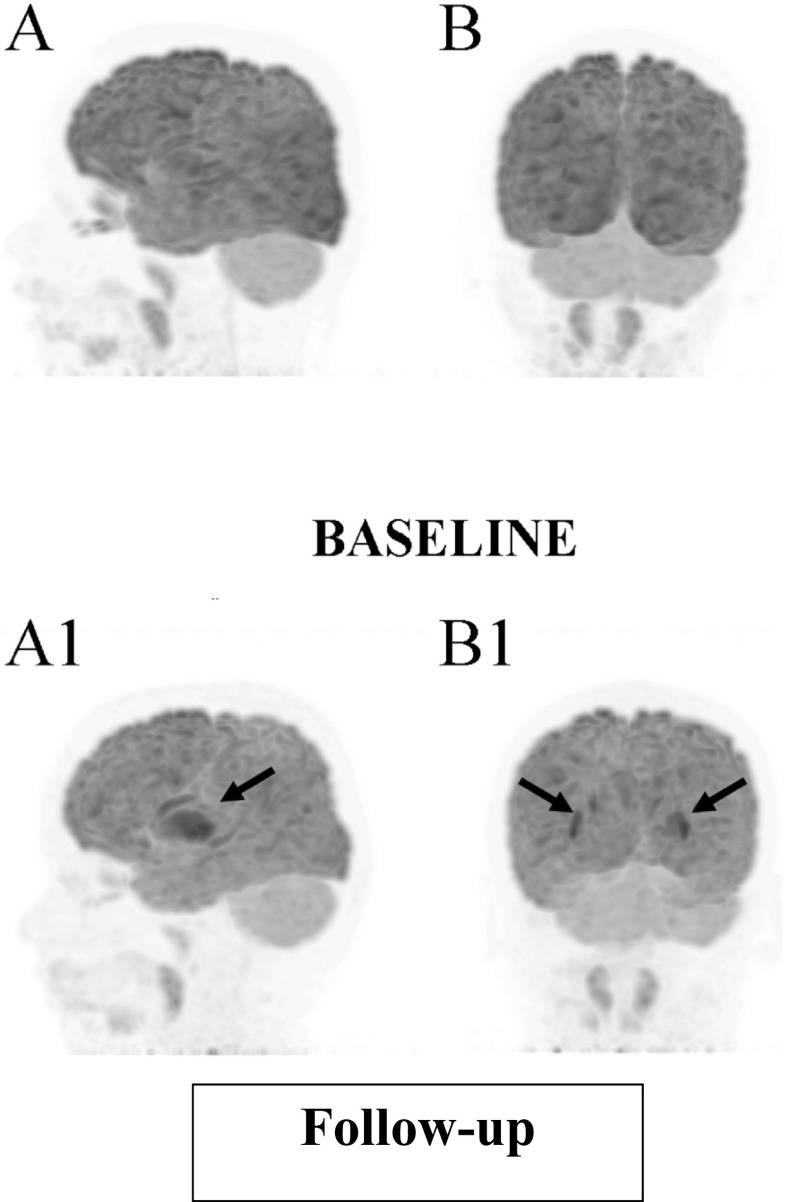
Maximum Intensity Projection of 18FDG PET. Baseline (before starting KD): **A** lateral view and **B** posterior view. Follow-up at 12 months after starting the KD: **A1** lateral view and **B1** posterior view – diffusely decreased activity in the brain cortex. Activity in the basal ganglia is relatively high (arrows)

Before starting the KD, regional glucose hypometabolism was observed bilaterally in the mesial temporal lobes, basal ganglia, and cerebellum (Fig. 1). After 12 months on the KD (1 month on the classic KD, 5 months on the MAD, and 6 months on the LGIT), 18F-FDG uptake decreased markedly and diffusely in the whole cerebral cortex with a relatively low reduction in basal ganglia (Figs. 2 and 3). Mean standardized uptake values (SUVs) at baseline (pre-KD) and at follow-up (KD) were: frontal lobes 8.6 and 6.1, temporal lobes 9.6 and 6.3, occipital lobes 9.5 and 6.0, parietal lobes 8.7 and 6.2, cerebellum 5.8 ad 4.3, and basal ganglia 7.6 and 6.2.

## Discussion

Almost one hundred years ago, Peterman, a paediatrician, noted improvements in his patients’ behaviour and cognitive functioning while being on the KD. He wrote: “In all the children treated with the ketogenic diet there was a marked change in character, concomitant with the ketosis, a decrease in irritability, and an increased interest and alertness; the children slept better and were more easily disciplined” (Peterman 1925). Soon after, studies on the therapeutic use of the KD concentrated on its anticonvulsant efficacy; its influence on behaviour was largely neglected. Some 80 years later, Pulsifer first sought to investigate the impact of KD on behaviour and intellect and described improvements in cognitive abilities (such as intellectual skills and concentration) in children on the KD that were unrelated to a reduction of drugs taken or clinical antiepileptic outcomes (Pulsifer et al. 2001).

This case report demonstrates the clinical efficacy of a 16-month ketogenic therapy in an autistic patient. Of note, the behavioural and intellectual improvements while on the KD occurred in a patient exhibiting pre-treatment PET imaging abnormalities of glucose hypometabolism in brain structures (i.e., amygdala, superior temporal sulcus, orbito-frontal cortex, fusiform gyrus, insula, anterior cingulated gyrus, and basal ganglia) that are involved in mediating social behaviours (Ventola et al. 2013; Zurcher et al. 2015). Furthermore, these behavioural improvements were maintained despite switching from classic KD to less restrictive diets such as MAD and then LGIT.

As far as KD therapy and ASD are concerned, very limited clinical information can be found in the literature. One study demonstrated that approximately 60% of autistic patients showed some improvement during KD therapy; however, only less than 10% of the improvement was clinically significant as measured by a 12-unit improvement on the CARS (Evangeliou et al. 2003). Of note, the authors applied a rather non-typical, intermittent KD protocol, with 4 weeks on the KD interrupted by 2 weeks diet-free intervals for 6 months.

One of the fundamental pathophysiological changes in autism is brain glucose dysregulation, which can manifest as a state of hypometabolism or hypermetabolism in the affected brain regions (Haznedar et al. 1997; Zurcher et al. 2015). Dysregulation of glucose metabolism may be related to impaired adenosine triphosphate production and/or storage, or to some mitochondrial defects. Interestingly, newer studies especially support the idea of a link between mitochondrial dysfunction and the behavioural phenotype of autism. This link may be related to decreased mitochondrial gene expression, decreased activity of electron transport chain complexes, or abnormal levels of peripheral markers of mitochondrial function (Napoli et al. 2014; Cheng et al. 2017). Individuals maintained on the KD become less dependent on glucose; as a result, energy requirements rely more on ketone bodies, which may explain some of the therapeutic effect of the KD in ASD (Frye and Rossignol 2014).

In the presented case, analysis of 18F-FDG PET images revealed the characteristic pattern of KD induced metabolic changes in the brain: a substantial and diffuse decrease of 18F-FDG uptake in the whole cerebral cortex with a relatively low reduction of activity in the basal ganglia. Semi-quantitative evaluation confirmed this finding by decreased SUVs. This pattern on 18F-FDG PET scans was previously reported in the patient with epilepsy who had been on KD for 2.5 months (Korsholm and Law 2013). Identification of focal metabolic disturbances in such cases is not feasible. In a recently published dual tracer PET quantification, the metabolic rate of glucose in the brain decreased by 20%, whereas acetoacetate increased 6-fold as early as four days after the introduction of the KD (Courchesne-Loyer et al. 2017).

It is unknown how exactly these metabolic changes in particular midbrain structures translate into clinical improvements. It is also unknown if glucose uptake alterations are required for clinical improvement. It is possible that this particular patient’s metabolic change, as displayed on 18F FDG PET images, merely enhanced the metabolism of neurons (e.g., by increasing oxidation of fatty acids) with consequent improvements in behaviour and intellect. Furthermore, one can speculate that ASD patients exhibiting glucose hypometabolism may benefit from reducing glucose supply into the brain and serving as a sole energy source for the brain.

The goal of personalized therapy is the ability to predict who would most benefit from its implementation. In the case presented here, glucose hypometabolism in discrete brain structures, as revealed by 18F-FDG PET, was observed at baseline and was further decreased after the patient continued on the KD for approximately 12 months. This decrease was potentially caused by a metabolic shift from the utilization of glucose to utilization of ketone bodies as a primary source of energy (Frye and Rossignol 2014; Courchesne-Loyer et al. 2017). Likewise, the patient’s behaviour and intellect improved when on the KD. Further exploration will study whether autistic patients exhibiting similar glucose hypometabolism in 18F-FDG PET would attain similar clinical benefits on the KD. Of note, other medical conditions (e.g., Alzheimer’s disease, mild cognitive impairment, or amyotrophic lateral sclerosis) associated with glucose hypometabolism are now thought to benefit from the KD or other approaches (e.g., ketone bodies, their metabolic precursors, or medium-chain-fatty acids) that affect glucose/lipid metabolism in a way that at least partially mimics the KD (Croteau et al. 2017; Koppel and Swerdlow 2017; Paoli et al. 2014).

## Abbreviations

ADHD: Attention-deficit hyperactivity disorder
ASD: Autism spectrum disorders
CARS: Childhood Autism Rating Scale
DSM-IV-TR: Diagnostic and Statistical Manual of Mental Disorders, 4th Edition, Text Revision
EEG: Electroencephalogram
18FDG PET: 18 fluoro-deoxyglucose positron emission tomography
KD: Ketogenic diet
LGIT: Low glycaemic index treatments
MAD: Modified Atkins diet
WISC-R: Wechsler Intelligence Scale for Children- revised
